# Fuel-cell breathalyser use for field research on alcohol intoxication: an independent psychometric evaluation

**DOI:** 10.7717/peerj.4418

**Published:** 2018-03-14

**Authors:** Jacob G. Sorbello, Grant J. Devilly, Corey Allen, Lee R.J. Hughes, Kathleen Brown

**Affiliations:** 1 School of Applied Psychology, Griffith University, Mt Gravatt, Queensland, Australia; 2 Queensland Police Service Academy, Queensland Police Service, Brisbane, Queensland, Australia; 3 School of Criminology, Griffith University, Mt Gravatt, Queensland, Australia; 4 Griffith Criminology Institute, Griffith University, Mt Gravatt, Queensland, Australia

**Keywords:** Breathalysers, Alcohol, Measurment, Reliability, Validity, Field trial

## Abstract

**Background:**

Several field studies have used fuel-cell breathalysers (FCB) to investigate the prevalence of alcohol intoxication. However, there is a lack of evidence evaluating the psychometric properties of these breathalysers outside of the forensic disciplines.

**Methods:**

The current research describes four studies designed that assess the reliability and validity of portable platinum FCBs for research on alcohol intoxication. Utilising the Alcolizer LE5 breathalyser and, to a lesser degree, the Lifeloc FC-20 and the Lion Intoxilyzer 8000, each study sampled patrons frequenting popular night-time entertainment districts with varying levels of alcohol intoxication.

**Results:**

Study one and two found excellent test-retest reliability and inter-instrument reliability for FCBs. Study three and four provided evidence to support the convergent validity of the two FCBs (the LE5 with the FC20), and with an evidential breathalyser (i.e., the Lion Intoxilyzer 8000; EB).

**Discussion:**

A 93–97% agreement rate between breathalyser readings was found across the four studies. Portable FCB are recommended as a reliable and valid instrument for research designs requiring quick alcohol intoxication estimations in large populations. Strategies to enhance reliable and valid readings are provided for field researchers.

## Introduction

Fuel-cell breathalysers (FCB) are screening instruments to obtain quick biological measures of alcohol intoxication (Dawe et al., 2002). Several studies have used breathalysers to investigate the prevalence of alcohol misuse (Moore et al., 2007; Thombs et al., 2010); and alcohol preloading in night-time entertainment districts (NED; Glindemann et al., 2006; Reed et al., 2011; Devilly, Allen & Brown, 2017). Despite their usage, these breathalysers have yet to be validated for field research. Field researchers seem to favour using breathalysers because the readings are more accurate than self-reported information by intoxicated individuals and observer ratings (Kraus et al., 2005; Rubenzer, 2011). With appropriate validation, FCBs could offer field researchers a reliable method to measure alcohol intoxication.

Alcohol intoxication is biologically measured by the individual’s blood alcohol concentration (BAC; Swift, 2003). This concentration refers to the amount (i.e., a percentage) of alcohol present in the blood stream and is measured in grams per decilitre of blood (e.g., a BAC of .05% equates to .05 grams of alcohol in every decilitre of blood; Dawe et al., 2002). Blood sampling collects true BAC; the non-invasive solution is to collect a breath sample with a portable breathalyser. This breath test provides an indirect approximated measurement of BAC (aBAC). In contrast to blood analysis, portable breathalyser testing has advantages that reduce administration time, provides quicker results, is less costly for the researcher, and can be conducted in the field (Smith, 2011; Dawe et al., 2002). There are some limitations with breath sampling because it can underestimate true BAC by 15% (Coyle, Field & Starmer, 2010; Kriikku et al., 2014).

Measurement error is a great concern for field researchers using breathalysers to record data on alcohol consumption. According to Gullberg (2006), three principle components influence aBAC measurement uncertainty: biological/sampling errors (e.g., breathing patterns); instrumental differences (e.g., device calibration), and traceability (i.e., inferences). Biological errors create the most uncertainty, but when combined these components can produce a cumulative effect impacting the precise measurement of aBAC. Researchers should control for degree of uncertainty when using aBAC readings by limiting measurement error (Gullberg, 2003; Gullberg, 2006; Coyle, Field & Starmer, 2010), and the more reliable an instrument, the smaller the measurement error will be.

A few studies have compared the variability of different breathalysers. Forensic evidence using drink driving data has demonstrated that FCB estimates were ‘forensically acceptable’ (Gullberg, 2003) and were highly correlated with evidential breathalysers (*r* = .978; EB) and blood sampling (*r* = .940; Zuba, 2008). Laboratory studies also found FCB estimates had strong associations with evidential (*r* = .91) and blood testing (*r* = .88; Schechtman & Shinar, 2011) devices. Despite the excellent relationship between breathalyser technologies, there still appears to be some measurement error between breathalyser readings, and it is unknown whether these findings could extend to survey-based field research. Drunk-drivers and laboratory participants may behave differently to patrons in the NED. There are also variations in environmental influences—such as temperature and humidity—which could influence further measurement error (Zuba, 2008). A timely field investigation could replicate these findings to validate FCB use for field researchers (Maner, 2016).

There are two issues to address here: (1) The supporting evidence for breathalyser use in field research requires external validation; and (2) this evidence must account for the breathalyser’s measurement error. Our methodological paper addresses this gap and assesses the reliability and validity of breathalysers for field research designs. Our aim was to comprehensively evaluate one type of measurement technology—a portable FCB. We present four methodological studies that test the psychometric properties of a portable platinum FCB (i.e., the Alcolizer LE5). In study 1 we predict excellent test-rest reliability of the breathalysers across time. We extend on this finding in study 2 and predict excellent inter-instrument reliability. In study 3 and 4 we aim to test the comparison breathalysers (LE5s) against a similar portable FCB (i.e., the Lifeloc FC20) and an EB (i.e., the Lion Intoxilyzer 8000). Based on previous forensic and laboratory evidence (e.g., Zuba, 2008), we predict excellent convergent validity between different breathalysers.

## General Method

### Participants

Two hundred and fifty individuals in total participated across four studies. Of the 42 participants used in study 4, 31 of them had also been used in study 2. This leads to 229 unique participants in this investigation of FCBs. All data were collected in the Australian state of Queensland from the night-time entertainment district (NED) of Brisbane Central Business District (CBD) and Fortitude Valley on Thursday, Friday, and Saturday nights between the hours of 9 p.m. and 4 a.m.

### Apparatus and materials

#### Approximated blood alcohol concentration

We used three different breathalysers to collect aBAC. The Alcolizer LE5 was the comparison portable breathalyser. This breathalyser was compared to the portable Lifeloc FC20 and the evidential Lion Intoxilyzer 8000. All breathalysers are manufactured and distributed internationally. Each breathalyser was calibrated to a 2,100:1 partition ratio (see Jones, 1990, for a more detailed explanation of partition ratios and blood or breath alcohol concentration). In this paper we use the acronym aBAC to stand for approximated Blood Alcohol Concentration. The breathalysers measure breath alcohol concentration (measured as grams of ethanol per 210 litres of breath), but then use an algorithm (multiplying by the partition ratio) to approximate Blood Alcohol Concentration (measured as grams of ethanol per decilitre [dL] of arterial blood).

#### Alcolizer LE5 (Study 1-4)

Four LE5 breathalysers (Alcolizer; Alcolizer Pty Ltd., Brisbane, Queensland, Australia) measured a participant’s aBAC. The breathalyser used an electro-chemical fuel-cell (platinum) to detect quantities between .000–.500 BAC with an accuracy of at least ± .01 at .100 BAC g/dL (generated from a breath sample, explained below). As a frame of reference, the legal drink driving limit in Australia is less than .05 BAC g/dL. The LE5 is certified by Australian standard 3547 and used by law enforcement agencies throughout Australia and South East Asia. In Queensland, Australia (the location of the research study) FCB readings are not admissible in legal proceedings. In other countries, however, breathalyser readings can be admitted in evidence to a court (e.g., California in the USA) although not all devices are treated equally during trial. Each breathalyser was recalibrated twice by the owning company during the research time.

#### Lifeloc FC20 (Study 3)

One Lifeloc FC20 (Lifeloc Technologies Inc., Osborne Park, WA, Australia) was used as the similar portable FCB to compare the LE5 against another similar breathalyser from a different manufacturer. This FCB detects BAC quantities from .000 to .600 with ±.005 at.100 BAC g/dL accuracy. The FC20 was adopted by law enforcement across the United States and other countries and certified under DOT/NHTSA standards. This breathalyser was recalibrated by the distributor before the night of sampling.

#### Lion Intoxilyzer 8000 (Study 4)

One Lion Intoxilyzer 8000 (Lion Breathalysers Australia, Hazelwood, NSW, Australia) was used as the evidential comparison. These breathalysers provide accurate aBAC readings for admission of legal proceedings to prove excessive intoxication (Workman Jr, 2014). The Intoxilyzer 8000 uses infrared spectroscopy at 3 and 9 µm to detect aBAC quantities between .000 and .500 g/dL. The Intoxilyzer 8000 is used by Police Services across Australia and certified to NMI standard R126 by the Australian National Measurement Institute and to International OIML R126 1998 (E) specifications. After each sample the breathalyser required at least 5–10 min to recalibrate and self-check between readings.

#### General procedure

A mixed between-within measure design was used to test the differences across breathalysers. Ethical approval was obtained through Griffith University’s Human Research Ethics Committee (ref: PSY/71/14/HREC and PSY/D&/15/HREC). Researchers were placed at taxi ranks, train stations, and outside nightclubs to engage with patrons as they arrived or departed the Brisbane NED. There was no exclusion criterion for level of intoxication. Participants were approached to complete a short survey and were offered a breathalyser test. If participants refused, but wanted to know their aBAC, they received a free breathalyser test as a community service. If the participant refused a breathalyser test and survey completion they were excluded from the study. Refusal rates in our original study (Devilly, Allen & Brown, 2017) were 14.67% (22 out of 150 people). We did not have anyone so intoxicated that we felt uncomfortable approaching them for a test. The LE5 and FC20 breathalysers were operated by a member of the research team. A Queensland Police Service (QPS) Officer operated the Intoxilyzer. After each testing procedure, participants were given an ID card with the research information and their specific number, which included a link to an accompanying website. At this website they could later contact the researchers and remove their consent and data when sober. Participant consent was obtained verbally (being in the city night time entertainment district with inebriated people) and the consent was also demonstrated by completing the short questionnaire.

### Diagnostics and selected analyses

SPSS v.24 (IBM, St. Leonards, NSW, Australia) and Statistica v13 (TIBCO Software, Palo Alto, CA, USA), were used for data screening and analyses. All analyses were tested at the standard *α* = .05 criterion for significance. Before the main analyses, variables were screened for clerical errors, missing values, and assumption violations.

In order to see whether two fuel-cell technologies are giving similar results, or whether a FCB is giving similar results to an evidentiary system, we need to calculate the instrument measurement error. This is approximated using the standard error of measurement (SEM). Using the current case, the SEM of a breathalyser is obtained by first computing the standard SEM between two administrations of the test. The actual formula (see Eq. (1a)) accounts for the test-retest reliability of the breathalyser (*r*_*xx*_): if the test-retest correlation was 1, then there would be no error in the instrument; if *r* = 0.9 there would be .1 missing from being a perfect measure (assuming the time to take the measurements was not a factor). This error estimate is then standardised using the standard deviation (usually from time 1; SD) of the test-retest data. This SEM then needs to be computed for two administrations, as demonstrated in Eq. (1b). This creates a standard error of the difference (SED) figure. This “SED describes the spread of the distribution of change scores that would be expected if no actual change had occurred. (A change) larger than 1 .96 would be unlikely to occur (*p* < .05) without actual change.” (p. 14; Jacobson & Truax, 1991). This leads us to Eq. (1c), where we can say that the difference between two tests is reliable (i.e., not due to measurement error; *Score*_*t*2_–*Score*_*t*1_) if it is greater than 1.96 times the SED (for 95% confidence). For 99% confidence we can say that difference between two tests is greater than measurement error if it is greater than 2.58 times the SED. This produces a reliable change index (RCI) whereby a participant’s score could fluctuate if there was measurement error between the instruments.

(1a) \begin{eqnarray*}& & SEM=SD\sqrt{1-{r}_{xx}}\end{eqnarray*}

(1b) \begin{eqnarray*}& & SED=\sqrt{2(SEM)^{2}}\end{eqnarray*}

(1c) \begin{eqnarray*}& & Scor{e}_{t2}-Scor{e}_{t1}\gt 1.96\;SED (95\text{%}\;\mathrm{CI}).\end{eqnarray*}

We used ClinTools (Devilly, 2007) for the calculation of the breathalysers’ RCI). If the difference between aBAC samples was *within a* 95% RCI then we assumed the difference between aBAC readings was not due to measurement errors, because there was no intervening effect that would warrant the difference being outside this interval. When the opposite was observed and the difference was greater than the 95% RCI—we assumed this difference between aBAC readings was affected by measurement error from the breathalysers.

## Study 1: Test-Retest Reliability

In study one we collected duplicate aBAC readings with four LE5 breathalysers. Our aim for study one was to assess FCBs reliability across time.

### Study 1 method

#### Participants

One hundred and forty-six participants (81 males and 65 females) with age range from 18–54 years ($\overline{x}=22.25$, *σ* = 5.20) participated in this study.

### Apparatus and materials

#### Blood alcohol concentration

Four LE5 breathalysers (2016; Alcolizer Pty Ltd., Brisbane, Queensland, Australia) were used to measure participant’s aBAC.

#### Survey

We used QuickTapSurvey (TableDabble, 2014) on two iPad tablets to record self-reported information. We asked demographic questions (age and gender) and the duration of time (i.e., in minutes) since their last alcoholic beverage. We also recorded the time taken between samples for a consistent test-retest period.

#### Procedure

Participants were approached to complete a short survey and offered a breathalyser test. Each participant was asked to wait for 30 s to check the accuracy of their reading, after which they then provided a second aBAC on the same breathalyser. A stopwatch application on the survey recorded the time between samples. The test re-test reliability period was established to a maximum of 120 s. After a second aBAC reading was conducted, the participants were thanked for their participation and given feedback on their aBAC reading.

### Study 1 results

Table 1 presents the descriptive statistics and correlations between samples with and without .000 readings. We analysed with and without .000 readings to reduce systematic bias in our results, because a zero rating would likely equal a 100% hit rate.

**Table 1:** Preliminary descriptive statistics and correlations of the test-retest of the four breathalysers with and without .000 readings.

	${\overline{x}}_{\mathrm{overall}}$	*σ*_overall_	First reading (*r*)	Second reading (*r*)	Time between (*r*)	Time since (*r*)
With .000^a^						
1. First reading (BAC)	.059	.049	–	–	–	–
2. Second reading (BAC)	.058	.048	.994^***^	–	–	–
3. Time between samples (s)	30.51	12.36	.412^***^	.424^**^	–	–
4. Time since last drink (min)	30.69	36.25	−.201^**^	−.201^**^	.022	–
Without .000^b^						
1. First reading (BAC)	.072	.044	–	–	–	–
2. Second reading (BAC)	.071	.044	.991^***^	–	–	–
3. Time between samples (s)	32.29	12.81	.326^**^	.343^**^	–	–
4. Time since last drink (min)	25.50	23.67	−.056	−.055	.067	–

**Notes.**

a *N* = 146.

b *N* = 119.

** *p* < .01.

*** *p* < .001.

The first and second readings, on average, differed by .001 aBAC and had an excellent test-retest correlation. The time between samples was associated with a larger aBAC, which meant participants with a higher aBAC took longer to provide a second sample—likely due to difficulty concentrating on the task. The time since their last drink was not significantly associated with aBAC. aBAC was analysed further between each breathalyser and sample (see Table 2).

**Table 2:** Descriptive statistics of test-retest by breathalyser without .000 readings.

	First reading (BAC)		Second reading (BAC)		Difference between samples (Absolute)		Correlation *r*_1st−2nd_	*N*
	$\overline{x}$	*σ*	$\overline{x}$	*σ*	${|}\overline{x}{|}$	|*σ*|		
Breathalyser 1	.086	.054	.083	.052	.004	.006	.992^***^	32
Breathalyser 2	.081	.045	.079	.045	.004	.005	.991^***^	27
Breathalyser 3	.053	.025	.053	.027	.003	.005	.980^***^	28
Breathalyser 4	.068	.041	.068	.041	.003	.004	.993^***^	32
**All**	**.072**	**.044**	**.071**	**.044**	**.004**	**.005**	.991^***^	**119**

**Notes.**

*** *p* < .001.

There was little difference between each first and second reading, and the test-retest correlation was excellent across all breathalysers. The differences between each first and second reading were calculated and transformed into an absolute value—because aBAC change could not be interpreted as a negative value in later analyses. Participant characteristics had limited influence on aBAC readings: the absolute difference between samples was not influenced by age (*r* =  − .07, *p* = .47), time since last drink (*r* =  − .10, *p* = .28), and there was a non-significant small effect between genders (*t*(117) = 1.14, *p* = .26, *g* = .21). To check if the breathalysers were reliable on average across test-retest we ran a One-Way Analysis of Variance (ANOVA) with the absolute difference between aBAC samples as the outcome variable. The absolute mean difference between test-retest was consistent across the breathalysers, *F*(3, 115) = .35, *p* = .79.

Next, we computed a 95% RCI for each breathalyser’s test-retest reliability. To calculate the number of aBAC samples that were impacted by measurement error we used the RCI of each breathalyser and the absolute aBAC difference between samples. If the absolute aBAC difference was less than the 95% RCI, then any difference between aBAC readings was judged as not impacted by measurement error and this was coded 1 = *No Change*. If the absolute aBAC difference was greater than the 95% CI then measurement error impacted the difference between aBAC readings and this was coded 2 = *Change*. Table 3 presents the RCI of each breathalyser, the absolute mean aBAC difference, frequency, and percentage of samples that were impacted/unaffected by measurement error.

**Table 3:** Reliable change of test-retest for each breathalyser.

	RCI	Change						
		${\overline{x}}_{\mathrm{1st}}$	*σ*_1st_	${\overline{x}}_{\mathrm{2nd}}$	*σ*_2nd_	${|}\overline{x}{{|}}_{\mathrm{difference}}$	|*σ*|_difference_	*N* (%)
Breathalyser 1	.0134	.165	.006	.142	.008	.023	.002	2 (6.30)
Breathalyser 2	.0112	.114	.025	.110	.001	.017	.007	2 (7.40)
Breathalyser 3	.0096	.087	.023	.088	.039	.014	.006	3 (10.70)
Breathalyser 4	.0095	.093	.055	.099	.047	.012	.002	4 (12.50)
**All**	**.0117**	**.138**	**.030**	**.133**	**.018**	**.017**	**.005**	**8 (6.72)**

	RCI	No Change						
		${\overline{x}}_{\mathrm{1st}}$	*σ*_1st_	${\overline{x}}_{\mathrm{2nd}}$	*σ*_2nd_	${|}\overline{x}{{|}}_{\mathrm{difference}}$	|*σ*|_difference_	*N* (%)
Breathalyser 1	.0134	.080	.052	.080	.052	.003	.003	30 (93.70)
Breathalyser 2	.0112	.079	.046	.077	.046	.003	.003	25 (92.60)
Breathalyser 3	.0096	.049	.022	.049	.022	.002	.002	25 (89.30)
Breathalyser 4	.0095	.064	.038	.063	.039	.002	.002	28 (87.50)
**All**	**.0117**	**.067**	**.041**	**.067**	**.042**	**.003**	**.003**	**111 (93.28)**

The RCI of each breathalyser found a high percentage of readings were consistent across the test-retest period. The readings that were impacted by measurement error had a higher mean aBAC than the no-change group. As a final analysis, we conducted a chi-square test to check if the test-retest change was different between breathalysers. There was no difference between the change/no-change groups of each breathalyser, *X*^2^ (3, *n* = 119) = .83, *p* = .84. This suggested that FCBs such as the LE5 produce similar aBAC readings across time. Indeed, the agreement rate of test-retests showed an overall 93.28% of samples were unaffected by measurement error.

## Study 2: Inter-Instrument Reliability

Our next step was to account for the measurement error between breathalysers. Measurement error could occur between the breathalysers because of manufacturing and calibration issues. Therefore, we assessed the inter-instrument reliability of these breathalysers. Our goal for study two was to test whether multiple FCBs produce similar readings.

### Study 2 method

#### Participants

Thirty-one participants provided aBACs on multiple instruments. Because participants’ characteristics had little influence on the psychometric properties of the FCBs, we decided not to record all demographic data in this and the following studies.

### Apparatus

#### Approximated blood alcohol concentrations

We used three LE5s from study one. QuickTapSurvey (2014; TabbleDabble Inc., Toronto, Ontario, Canada) on two iPad tablets to record the aBAC readings.

#### Procedure

Participants were approached to take part in multiple breathalyser tests to check the reliability of the breathalysers. Each participant provided a sample to each of the three breathalysers in an alternating number system, within less than 30 s between the samples. The alternating number system rotated the order in which the breathalysers were used, but this occasionally went out of synchronicity (due to field trial issues, such as inebriated people saying they wanted the blue coloured breathalyser last or they wanted a specific researcher to breathalyse them with all the breathalysers).

### Study 2 results

Table 4 presents the descriptive statistics and correlations of aBAC readings between LE5s’. Three participants were excluded because they provided .000 aBAC readings. Each breathalyser found similar aBAC readings and excellent correlation.

**Table 4:** Descriptive Statistics and Correlations of aBAC readings.

	$\overline{x}$	*σ*	1.^a^	2.^a^	3.^a^
1. Breathalyser 1 BAC	.091	.040	–	–	–
2. Breathalyser 2 BAC	.088	.039	.994^***^	–	–
3. Breathalyser 3 BAC	.090	.040	.993^***^	.990^***^	–

**Notes.**

*N* = 28. ${\overline{x}}_{\mathrm{overall}}=.090,{\sigma }_{\mathrm{overall}}=.039$.

a Pearson’s moment correlations (*r*).

*** *p* < .001.

We ran a Repeated Measures ANOVA to check the differences within the subjects’ aBAC measurements. Mauchely’s test of sphericity was not violated and there was a significant difference with a small effect size in the ‘within subjects’ factor of aBAC readings (*F*(2, 54) = 6.84, *p* < .01 ${\acute {\eta }}_{p}=.20$). This suggested that 20% of the variance was attributed to the difference between the breathalyser aBAC readings.

Despite this small variance between the breathalysers’ readings, we placed more importance on the 95% RCI established in study 1 (i.e., RCI = .0117) to account for the measurement error between breathalyzers. We used the same coding specification from study 1 to recode the absolute difference between aBAC readings and calculate the number of samples that were impacted by measurement error. Table 5 displays the mean and standard deviation of the absolute aBAC difference between breathalyzer readings, as well as the frequency and percentage of samples impacted/unaffected by measurement error.

**Table 5:** Reliable change between each breathalyser.

	RCI	Change			No change		
		${|}\overline{x}{{|}}_{\mathrm{difference}}$	|*σ*|_difference_	*N* (%)	${|}\overline{x}{{|}}_{\mathrm{difference}}$	|*σ*|_difference_	*N* (%)
Breathalyser 1–2	.0117	.013	.002	2 (7.14)	.004	.003	26(92. 86)
Breathalyser 1–3	.0117	–^a^	–	1 (3.57)	.003	.003	27 (96.34)
Breathalyser 2–3	.0117	.017	.001	2 (7.14)	.003	.003	26 (92.86)
**All Comparisons**	**.0117**	**.014**	**.002**	**5 (5.95)**^b^	**.003**	**.003**	**79 (94.05)**^c^

**Notes.**

a aBAC was high for this reading (Breathalyser 1 aBAC = .193; Breathalyser 3 aBAC = .179).

b $\overline{x}=.143$, *σ* = .044.

c $\overline{x}=.086$, *σ* = .037.

Five samples were impacted by measurement error. These readings had a higher overall mean than the unaffected readings. Ninety-four percent of the readings were reliable between replica FCBs.

## Study 3: Convergent Validity with a Similar Breathalyser

After establishing reliability indices, we moved to assess the validity of the LE5 FCB by comparing it to different breathalyser’s aBAC reading. The goal of study three was to test the convergent validity of similar portable platinum FCBs.

### Study 3 method

#### Participants

Forty-two participants provided aBAC readings.

### Apparatus and materials

#### Approximated blood alcohol concentration

We used the same three LE5s in study two to collect the participants’ aBAC. To compare the qualities of the LE5 we used the FC20 breathalyser.

#### Procedure

Participants were approached for a breathalyser test. Each participant was randomly assigned to provide a sample to one of the three LE5 breathalysers or the FC20 breathalyser first. After each participant provided their first aBAC sample they were then re-tested with the alternative breathalyser for a second BAC reading. For example, if the participant provided their first aBAC sample to a LE5 breathalyser, their second aBAC sample was tested on the FC20. Readings were taken within 30 s of each other.

### Study 3 results

Eight participants were excluded because they provided .000 aBAC readings. The FC20 recorded similar aBAC readings ($\overline{x}=.074$, *σ* = .042, *n* = 34) to the LE5 ($\overline{x}=.073$, *σ* = .041, *n* = 34). There was minimal difference between the different FCB readings (${\overline{x}}_{\mathrm{difference}}=.002$, *σ*_difference_ = .006) and the relationship between aBAC readings was excellent (*r* = .991, *p* < .001). To check for order and sampling effects we ran a Mixed 2 (aBAC: FC20, LE5) × 2 (Order of testing: FC20 used first or second) ANOVA. There was no significant between-subjects main effect for order of testing *F*(1, 32) = .39*p* = .54, and no interaction with aBAC readings *F*(1, 32) = 1.75, *p* = .20. The within subjects effect of aBAC was approaching significance with a small effect size, *F*(1, 32) = 3.57, *p* = .07, ${\acute {\eta }}_{p}=.10$. Ten percent of the variance between different fuel-cell aBAC readings was possibly due to instrumental differences.

Consistent with the two previous studies we used the 95% RCI (i.e., .0117) established from the test-retest of the LE5. We used the same coding specification from the previous studies to recode the absolute difference between the FC20s’ and LE5s’ aBAC readings, and calculate the number of samples that were impacted by measurement error. Thirty-two samples displayed no difference between different FCB readings^1^ 1Change- FC20 ($\overline{x}=.140$, *σ* = .012) LE5 ($\overline{x}=.125$, *σ* = .011); No Change- FC20 ($\overline{x}=.071$, *σ* = .040) LE5 ($\overline{x}=.070$, *σ* = .040).(94.12%; ${|}\overline{x}{{|}}_{\mathrm{difference}}=.005$, |*σ*|_difference_ = .003), while the difference between 2 participants’ readings were impacted by measurement error (${|}\overline{x}{{|}}_{\mathrm{difference}}=.012$, |*σ*|_difference_ = .000; 5.88%). The two samples impacted by measurement error recorded a higher aBAC with both breathalysers.

## Study 4: Convergent Validity with an Evidential Breathalyser

Our final step was to assess the LE5 FCBs against an EB. We replicated our design from study three, except our comparison breathalyser was the Lion Intoxilyzer 8000. Our goal in study four was to assess the convergent validity between a FCB and an EB.

### Study 4 method

#### Participants

Forty-two participants provided aBAC readings. Study 2 and study 4 participants were collected on the same night.

### Apparatus and materials

#### Approximated blood alcohol concentration

We compared the same three LE5s to the Intoxilyzer 8000.

#### Procedure

Participants were approached for a breathalyser test. Each participant was randomly assigned to provide a reading to the Intoxilyzer or LE5 breathalyser first. The Intoxilyzer was situated on the roof of a QPS vehicle. Each participant provided three samples to the LE5 breathalysers while the Intoxilyzer recalibrated between readings. LE5 sampling followed the alternating number system as used in study 2. Similar to the previous study procedure, each participant was re-tested with the alternative breathalyser for a comparison reading. In total, each participant provided four aBAC readings: one to the Intoxilyser and three to each LE5.

### Study 4 results and discussion

Six participants were excluded because they provided .000 aBAC readings. The Intoxilyzer recorded similar aBAC readings ( $\overline{x}=.080$, *σ* = .044, *n* = 36) to the mean BAC of the LE5s’ ($\overline{x}=.078$, *σ* = .044, *n* = 36). There was little difference between the FCB readings and the EB readings (${\overline{x}}_{\mathrm{difference}}=-.001$, *σ*_difference_ = .005) and the relationship between aBAC measurements was excellent (*r* = .995, *p* < .001). To check for order and sampling effects we ran a Mixed 2 (aBAC: Intoxilyzer, LE5) × 2 (Order of testing: Intoxilyzer used first or second) ANOVA. There was no significant between-subjects main effect for order of testing *F*(1, 34) = .53*p* = .47 and no interaction with aBAC readings *F*(1, 34) = .05, *p* = .94. The within subjects effect of aBAC was non-significant, *F*(1, 34) = 2.16, *p* = .15, suggesting little difference between the two technologies.

To conclude our validity analysis, we calculated the absolute difference between all Intoxilyzer readings and LE5 readings. The 95% RCI (i.e., .0117) based on the test-retest of the LE5 in study 1 was used to conservatively estimate the measurement error for both types of breathalysers. We used the same coding specification from the previous studies to recode the absolute difference between the Intoxilyzer and LE5s’ aBAC readings, and calculate the number of readings that were impacted by measurement error. One reading between the EB and FCB was impacted by measurement error (2.86%; Intoxilyzer aBAC = .199; LE5 aBAC = .184), while 35 samples were unaffected by measurement error between the different measurement methods (97.14%; ${\overline{x}}_{\mathrm{Intox}}=.076$, *σ*_Intox_ = .039; ${\overline{x}}_{\mathrm{LE5}}=.075$, *σ*_LE5_ = .039; ${|}\overline{x}{{|}}_{\mathrm{difference}}=.003$, |*σ*|_difference_ = .003).

## General Discussion

We tested the psychometric properties of portable FCBs for field research over four studies by accounting for the measurement error between aBAC readings. Study 1 found excellent test-retest reliability for LE5 FCB across a short time interval and study 2 demonstrated excellent inter-instrument reliability between breathalysers. After reliability indices were established, we evaluated two types of breathalyser using the same technology (fuel-cell) and one evidential system using infrared spectroscopy. Study 3 and study 4 demonstrated impressive convergent validity with the FC20 (a similar portable FCB) and to the Intoxilyzer 8000 (an evidential infrared spectroscopy breathalyser). Our results found a 93%–97% agreement between aBAC readings over the 4 studies.

Study 1 found duplicate FCB aBAC readings were exceptionally reliable after accounting for measurement error. The difference in aBAC readings was not influenced by the age or gender and was consistent with past research (Devilly, Allen & Brown, 2017). Measurement error was largely influenced by higher aBAC readings (>.100 aBAC) which emerged across our four studies and is also evident in the forensic literature with different breathalysers (Gullberg, 2003; Gainsford et al., 2006; Schechtman & Shinar, 2011). Error at higher aBAC ranges is understandable given FCBs are predominantly designed to screen for intoxication around the legal driving limit (i.e., .050 BAC g/dL for Australia; .080 BAC g/dL for the USA and England); and the standards governing their manufacture specify greater accuracy between .050–.100 BAC g/dL in Australia, where the testing took place (Standards Australia, 1997). However, four readings in study 1 were impacted by measurement error—two from breathalyser 3 and two from breathalyser 4—that were below .100 aBAC. Perhaps these duplicate readings were impacted by external sampling or instrument errors (Gullberg, 2006). That said, the FCBs were consistently reliable across time.

Study 2 demonstrated excellent inter-instrument reliability. Similar to study 1, the five impacted readings were at a higher aBAC range. We think breathing patterns (a biological/sampling error; Gullberg, 2006) may have contributed to the uncertainty between these samples. Breath volume—which can be influenced by smoking, medical problems and physical lung volume—impacts the aBAC reading because a participant’s sample may not contain the adequate volume for the machine to test (Gullberg, 2006; Hlastala & Anderson, 2007; Black, 2017). We instructed participants to provide three breath samples within a 30 s delay, which may have influenced the sampling error. For instance, the error between breathalysers 1–3 seemed to occur because the continuous exhalation of the participant’s breath across three different breathalysers reduced the available detectable alcohol molecules from the first breathalyser reading to subsequent readings. This error became more pronounced at higher aBAC readings. Researchers can mitigate this sampling error by allowing the participant to return to a normal breathing pattern before conducting follow-up readings.

Study 3 and 4 found the comparison FCB aBAC readings were valid with readings from different breathalysers. Consistent with study 1 and 2, measurement error occurred at higher aBAC readings. Consistent with past research (Zuba, 2008; Schechtman & Shinar, 2011), our results found a strong relationship between FCB and EB aBAC readings. Gullberg (2003) and Zuba (2008) found similar error variation at higher aBAC readings against multiple breathalysers from different manufacturers. We expected more difference between the EB and FCB readings, because prominent instrumental differences (i.e., infrared vs. fuel-cell detection) could have contributed to more measurement error (Gullberg, 2006). Perhaps our procedure provides an explanation, which differed slightly between study 3 and 4. The EB required at least a five-minute recalibration and self-checking period between readings, while the FCBs had a short recalibration window. This allowed some participants in study 4 enough time to return to a normal breathing pattern which reduced the impact of biological sampling errors (Gullberg, 2006). Overall, our validity evaluation found little difference in aBAC readings between fuel-cell and infrared spectroscopy based EBs.

### Implications for field research

To our knowledge this research constitutes the first non-forensic and independent investigation on the psychometric properties of portable breathalysers for field research. Previous investigations (e.g., Gullberg, 2003; Zuba, 2008; Schechtman & Shinar, 2011; Leonard, 2012) analysed the precision and reliability of various breathalysers in forensic and laboratory studies. We have built upon this research by conducting studies which are generalisable to the population field researchers intend to sample. Our findings imply that fuel-cell technology is reliable and valid for researchers who plan to use portable breathalysers for future investigations of alcohol intoxication in large representative populations.

With that said, future field researchers should consider which breathalyser technology to employ in their research designs. We found the FCBs much quicker, easy to administer and cheaper than the evidential system. FCBs would be useful for large-scale alcohol research that require quick aBAC assessments—e.g., research establishing baseline intoxication trends in NEDs. However, it has been argued that FCBs will have greater variation from environmental conditions than infrared systems (Zuba, 2008). We recommend using infrared systems with smaller sample sizes and where researchers are seeking to test a new FCB, while using a different technology as a comparison. It is important to note, however, that an EB is more expensive, requires training to operate and takes longer to administer and test aBAC. In contrast, the EB is generally less susceptible to mouth alcohol (Leonard, 2012).

Mouth alcohol is a large threat to aBAC validity. This is the presence of alcohol that remains in the lining of the mouth, which exaggerates the aBAC (Black, 2017). Fuel-cell detectors are confounded by mouth alcohol, because the detector cannot differentiate between mouth or breath alcohol (Leonard, 2012). In general, mouth alcohol has a greater influence on individuals with low BAC, because it has an inversely proportional dissipation rate with actual BAC (Gullberg, 1992). Individuals who also rinse alcohol around their mouth (e.g., wine drinkers) can influence higher mouth alcohol aBAC (Wigmore & Leslie, 2001). If future researchers suspect mouth alcohol is inflating aBAC they should corroborate their own observations of the participant and the time since their last drink, before re-testing the participant.

Mouth alcohol can produce a 20% decrease between first and second aBAC readings (Sterling, 2012). Our readings of error across the four studies demonstrated^2^ 2The average percentage difference for the first and second readings at measurement error: Study 1—3.28% decrease; Study 2—7.26% & 9.10% decrease, 6.53% increase; Study 3–8.28%–9.13% decrease; Study 4—3.95–9.82% decrease.a small average decrease between the first and second aBAC readings, but were much lower than Sterling’s (2012) 20% inference. While we are not certain mouth alcohol entirely explains this decrease, the second aBAC readings often decreased—as shown by Sterling (2012). However, this decrease could be attributed to irregular breath patterns, error at high aBAC readings and other external influences (Gullberg, 2003). Researchers could reduce the impact of mouth alcohol by setting a minimum 10-minute exclusion period since their last drink before participants are ready for breath sampling (e.g., Leonard, 2012) or by having water available for mouth rinsing.

Future researchers should also consider their exclusion criteria when sampling participants, being respectful of the participant’s informed consent (Aldridge & Charles, 2008). A representative sample of any NED will inevitably include heavily intoxicated individuals and those who are uncomfortable providing aBAC samples. Considering we encountered greater measurement error at higher aBAC ratings, one could argue that omitting heavily intoxicated individuals might be an option. However, not all high aBAC readings were impacted by measurement error, which suggests external influences fluctuated aBAC readings (Gullberg, 2006). We argue that participants should not be excluded at the first-point of contact based on experimenter observation because intoxication impacts all individuals differently and this approach will bias the sample (see Devilly, 2018). Understandably, if the participant is not able to proceed with the research—because of extreme intoxication and cognitive impairment—then it would be reasonable for the researcher to terminate the testing and count this as a ‘refusal’ or ‘omission due to impairment’ in the study attrition data. In our study we did not have anyone so intoxicated that they could not complete a breath test, although we had people who walked past us when offered the test.

### Limitations

Our four-part study was not without its limitations. We used a RCI derived from the test-retest reliability of the LE5 and extended this index to estimate the measurement error between different breathalysers. The range of measurement error (i.e., RCI = .0117 aBAC) was better than ±.5 of a standard drink—if we assume that 1 standard drink roughly equals .025 BAC g/dL. Still, we concede the evidence for the FCBs convergent validity must be viewed as the ‘best estimate’, because the test-retest reliability of the FC20 and the Intoxilyzer were unknown at the time of analysis. Efforts to contact the manufacturers of the FC-20 were unsuccessful. Future researchers could establish the test-retest for all comparison breathalysers. A second limitation concerns the inference of aBAC readings in place of blood-sampled BAC. This inference is problematic because aBAC readings were argued to underestimate true BAC by 15% on average (Coyle, Field & Starmer, 2010; Kriikku et al., 2014). We did not compare aBAC to blood analysis taken from blood samples, because we conducted field testing of intoxicated patrons in the night-time entertainment districts—an unsuitable environment to collect blood samples from inebriated participants.

### Conclusion

Overall, our study featured a strong design and clear analyses to assess the psychometric properties of fuel-cell breathalyser technology. We first established the test-retest reliability of the instrument (i.e., the LE5) to check reliability across time. To evaluate the psychometric properties of these breathalysers we accounted for measurement error between aBAC readings and validated the breathalyser against itself, a similar portable FCB (i.e., FC20), and an EB (i.e., Intoxilyzer 8000)—three breathalysers endorsed by rigorous domestic and international measurement standards. Each breathalyser was regularly calibrated by the distributors to ensure accurate readings were obtained. Our samples were collected from patrons that frequented the NED, which established external validity of these instruments for field research designs. In sum, our results provided excellent reliability and validity for fuel-cell technology and we recommend the use of these breathalysers for field studies of alcohol intoxication.

##  Supplemental Information

10.7717/peerj.4418/supp-1 Supplemental Information 1 Data files and output from spss

10.7717/peerj.4418/supp-2 Supplemental Information 2 Participant questionnaire

## Additional Information and Declarations

### Data Availability

The following information was supplied regarding data availability: The raw data is provided as a Supplemental File.
