# Supplementary figures and images for: Fuel-cell breathalyser use for field research on alcohol intoxication: an independent psychometric evaluation
Source: PeerJ. 2018 Mar 14;6:e4418. doi: 10.7717/peerj.4418 (PMC5857179; doi:10.7717/peerj.4418)

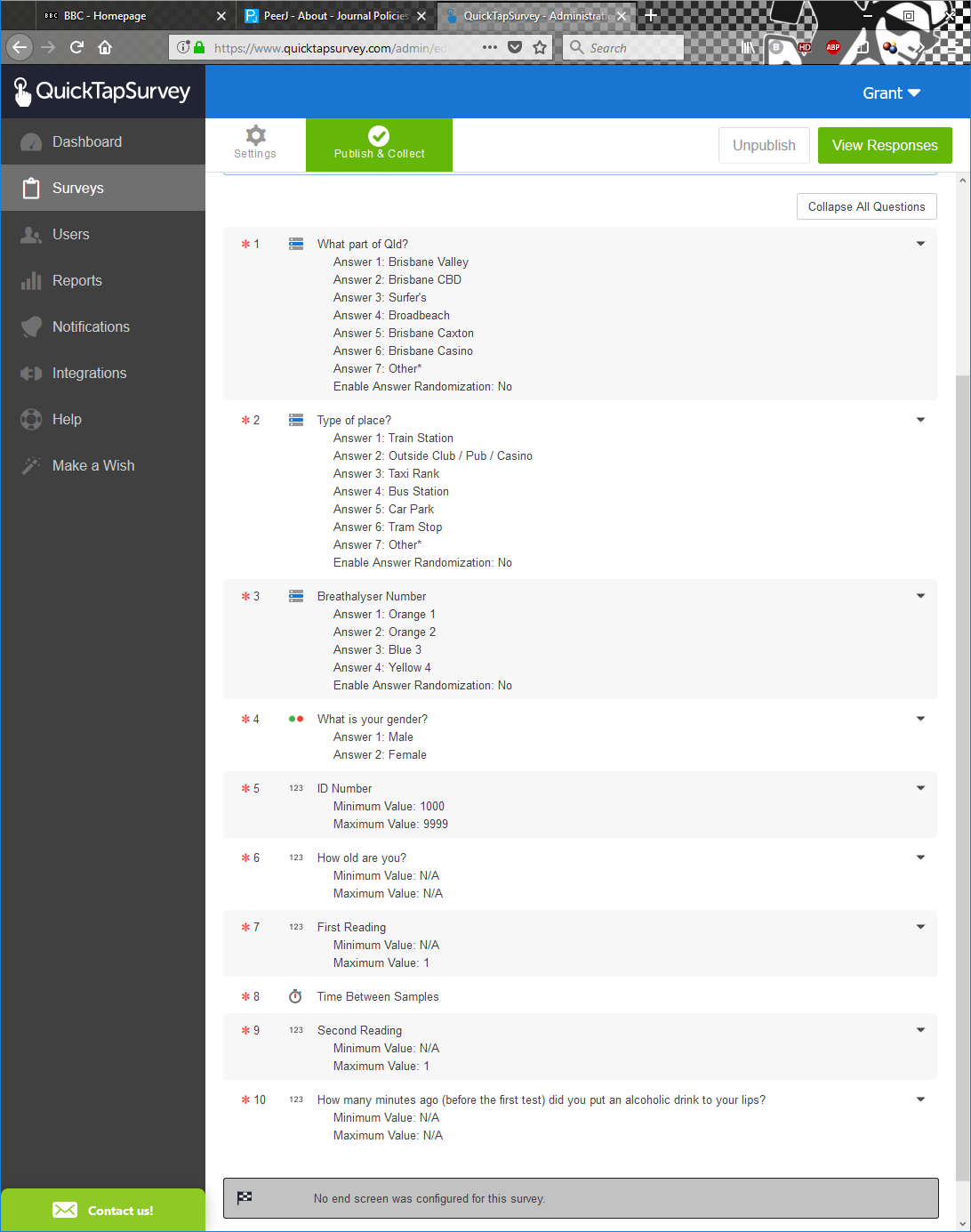

Supplement: Supplemental Information 2 [file peerj-06-4418-s002.docx]
